# A security testing mechanism for detecting attacks in distributed software applications using blockchain

**DOI:** 10.1371/journal.pone.0280038

**Published:** 2023-01-20

**Authors:** Abdullah Algarni, Abdulaziz Attaallah, Fathi Eassa, Maher Khemakhem, Kamal Jambi, Hosam Aljihani, Khalid Almarhabi, Faisal Albalwy

**Affiliations:** 1 Computer Science Department, King Abdulaziz University, Jeddah, Saudi Arabia; 2 Department of Computer Science, College of Computer Science and Engineering, Taibah University, Madinah, Saudi Arabia; 3 Department of Computer Science, College of Computing at Alqunfudah, Umm Al-Qura University, Makkah, Saudi Arabia; 4 Division of Informatics, Imaging and Data Sciences, University of Manchester, Manchester, United Kingdom; University of Pisa, ITALY

## Abstract

Distributed software applications are one of the most important applications currently used. Rising demand has led to a rapid increase in the number and complexity of distributed software applications. Such applications are also more vulnerable to different types of attacks due to their distributed nature. Detecting and addressing attacks is an open issue concerning distributed software applications. This paper proposes a new mechanism that uses blockchain technology to devise a security testing mechanism to detect attacks on distributed software applications. The proposed mechanism can detect several categories of attacks, such as denial-of-service attacks, malware and others. The process starts by creating a static blockchain (Blockchain Level 1) that stores the software application sequence obtained using software testing techniques. This sequence information exposes weaknesses in the application code. When the application is executed, a dynamic blockchain (Blockchain Level 2) helps create a static blockchain for recording the responses expected from the application. Every response should be validated using the proposed consensus mechanism associated with static and dynamic blockchains. Valid responses indicate the absence of attacks, while invalid responses denote attacks.

## Introduction

Distributed systems have recently superseded centralised systems. This is sensible because distributed systems have superior reliability, availability and incremental scaling potential. Component-based distributed systems deploy constituent components across multiple hosts and regions [1,2]. Distributed software applications deliver services over the internet. Validating the security of such applications is challenging because they are exposed to different kinds of attacks [3–6]. Malicious users are growing increasingly influential despite substantial improvements in internet security tools. Hackers typically target financial transactions. Specifically, they seek to redirect remittances or deposits to their accounts and access victims’ accounts.

Hackers also target the management infrastructure of well-known companies, news providers and other important websites to disrupt functioning. Most hackers use newer forms of attacks that are growing at an unprecedented rate. Blockchain is considered one of the most promising security technologies and might be a potential solution to such problems.

It is critical to use secure channels to share valuable information since it is not always possible to ensure the trustworthiness of individuals or groups, which is especially the case when working with unfamiliar people or entities. Therefore, the best-designed blockchain systems comprise data protection solutions to safeguard data against attacks. All blocks or records are secured using cryptography, which uses a private key and an individual’s digital signature for every block. Hence, blockchain advantages can be used for security testing. An assessment of the policies enforced using blockchain and the resulting efficacy shows that blockchain fulfils three primary security criteria: confidentiality, availability and integrity.

Several scientific publications have considered the application of blockchain technology and used specific criteria to address data-related issues, such as dependability, security and trust [7]. Parizi et al. [8] performed an empirical evaluation of automated security testing tools to detect security vulnerabilities using blockchain technology and smart contracts. Furthermore, another platform comprises transactive Internet of Things (IoT) blockchain applications, where testing is based on combinations of the test network and user patterns based on a custom domain-specific language to enhance features. These features concern the management, development, deployment and fault tolerance of the IoT blockchain [9].

In addition, Sharma et al. [10] devised the DistBlockNet distributed IoT network architecture using blockchain. It provides scalability, flexibility and communication security between different smart device categories. Smith [7] discussed an IoT blockchain case study and evaluated security and privacy criteria to ensure safe data management for smart cities.

However, using blockchain technology for security testing is a relatively new but popular topic. Blockchain studies in this domain are in their early stages and require further investigation concerning undiscovered security vulnerabilities in software or systems. This paper proposes a blockchain-based framework to support software security testing. We present the framework architecture and describe a proof-of-concept implementation for the proposed framework.

## Materials and methods

### Related work

Several studies [11–16] have suggested using blockchain technology to build a security testing mechanism and detect attacks, especially those targeting distributed software applications. The review starts with a survey conducted by Xie et al., comprising 56 studies [17]. The authors performed a systematic study on the security threats concerning blockchain and presented a survey about real-world attacks by assessing popular blockchain systems. They then evaluated security enhancement solutions for blockchain and provided recommendations for future research on this topic.

Smith [7] presented another survey with 48 studies. This research covered the current applications, research and critiques of blockchain. The aim was to highlight its benefits and limitations. The authors suggested three criteria as success predictors for blockchain-based data management projects: dependability, security and trust.

Meng et al. [18] presented a review on the intersection of Intrusion Detection Systems (IDSs) and blockchains, which included 39 studies. They first provided the background of intrusion detection and blockchain, followed by a discussion on blockchain applicability for intrusion detection and the identification of open challenges concerning this domain. They concluded that blockchains might influence IDS improvements; however, they claimed that the technology could not address all IDS issues.

Moreover, Roy et al. [19] explained the importance of blockchain for IoT security, privacy and management. They presented an overview of the literature concerning current progress, IoT security enhancements, scope and limitations. They concluded with recommendations for future research and suggested improvements.

Liang et al. [20] proposed a blockchain-based data provenance framework for the cloud. They identified various challenges concerning security and performance in adopting the proof-of-work (PoW) consensus protocol within this framework. The authors offered an overview of the unique design challenges and opportunities for developing proof-of-stake protocols for data attribution on a cloud-based platform.

Parizi et al. [8] presented a comprehensive empirical evaluation of automated security analysis tools for detecting vulnerabilities. They tested these tools using 10 real-world smart contracts; these tests addressed vulnerability and accuracy. They concluded that using IoT and smart contracts enhances network security using blockchain. The authors asserted that these two aspects have immense potential for the future of IoT security.

Sharma et al. [10] proposed a DistBlockNet security architecture. It is a new secured distributed IoT network architecture consisting of an SDNbase network based on blockchain. The authors asserted that security systems must automatically adapt to a threat landscape without administrator intervention. It eliminates the need to review and apply thousands of recommendations and opinions manually.

The authors also evaluated the performance of the proposed model and compared it with the existing model based on several metrics. The results indicated that DistBlockNet could detect IoT network attacks in real time with little performance overhead. Performance evaluation was conducted regarding the scalability, defensive effects, accuracy rates and performance overhead of the proposed model. The researchers claimed that the results indicated the efficiency and effectiveness of the DistBlockNet model. Moreover, they asserted that they fulfilled the design requirements with minimal overhead.

Gu et al. [21] proposed a consortium blockchain framework comprising a chain guarded by a consortium of members while users operate on a shared public chain. The results indicated that the proposed method could achieve high detection accuracy with lower false-positive and false-negative rates in a limited time. Another study by Pourmajidi and Miranksyy [22] proposed a blockchain-based logging system named ‘Logchain.IT’.

The system collected logs from different providers and prevented tampering by sealing them cryptographically and adding them to a hierarchical ledger. The authors claimed that their proposal provided an immutable platform for log storage. The technique addressed managing the extensive logs generated by cloud solutions where several tampering possibilities existed.

The abovementioned works indicate that solutions have been proposed for several aspects; however, the challenges concerning attack detection in distributed software application environments remain because these studies offer domain-specific solutions. In addition, no study has attempted to use software security testing functionality to enhance attack detection systems by exploring the benefits of blockchain technology. This study aims to provide a new and promising solution in this direction.

### System architecture

**Fig 1:**
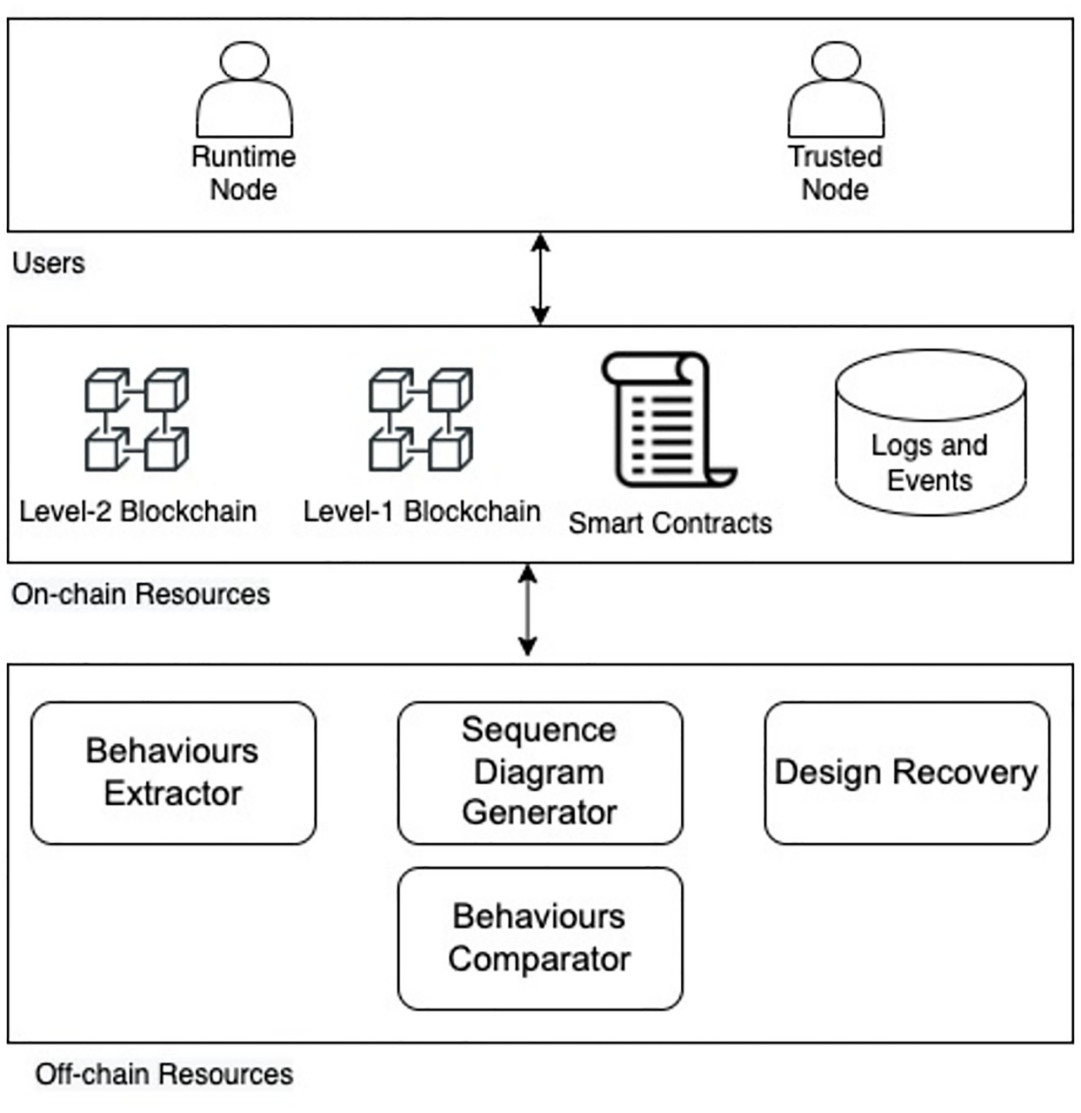
The system architecture.

The proposed blockchain-based framework for a security testing system includes several integrated modules to detect malware attacks on software programs. The modules include users, on-chain resources and off-chain resources. Fig 1 depicts the overall system architecture.

- Users
  - Trusted Node: A software developer or any other actor that owns a software source.
  - Run-Time Node: A software user or any actor that has the software execution file.
- On-chain resources
  - Blockchain Level 1: Provides a decentralised infrastructure for storing and managing trusted software behaviour.
  - Blockchain Level 2: Provides a decentralised infrastructure for storing and managing run-time software behaviour.
  - Smart Contracts: These are used to provide system functionalities, such as node registration, trusted software behaviour management and run-time software behaviour management.
  - Logs and events: Logs and events are created by smart contracts for all system transactions.
- Off-chain resources
  - Sequence Diagram Generator: A tool used to convert the software source code to a sequence diagram.
  - Behaviours Extractor: A tool used to convert the sequence diagram from the graphical and text-based representation to a JSON file representation.
  - Design Recovery: A tool used to extract the sequence diagram during the execution time to a JSON file representation.
  - Behaviours Comparator: A tool used for comparing the behaviour obtained from Behaviours Extractor and the behaviour obtained from Design Recovery.

### Smart contracts

Three smart contracts were written to provide system functionalities: Registration Smart Contract, Trusted Behaviour Smart Contract and Run-time Behaviour Smart Contract. Table 1 shows the system smart contracts’ main function.

**Table 1:** System smart contracts’ main functions.

#	Function	Descriptions
**1**	authorizeNode	Responsible for granting authorisation for nodes
**2**	unauthorizeNode	Responsible for revoking authorisation for nodes
**3**	storeTrustedEvent	Responsible for storing and managing trusted software behaviour
**4**	storeRunTimeEvent	Responsible for storing and managing run-time software behaviour

#### Registration smart contract

**Fig 2:**
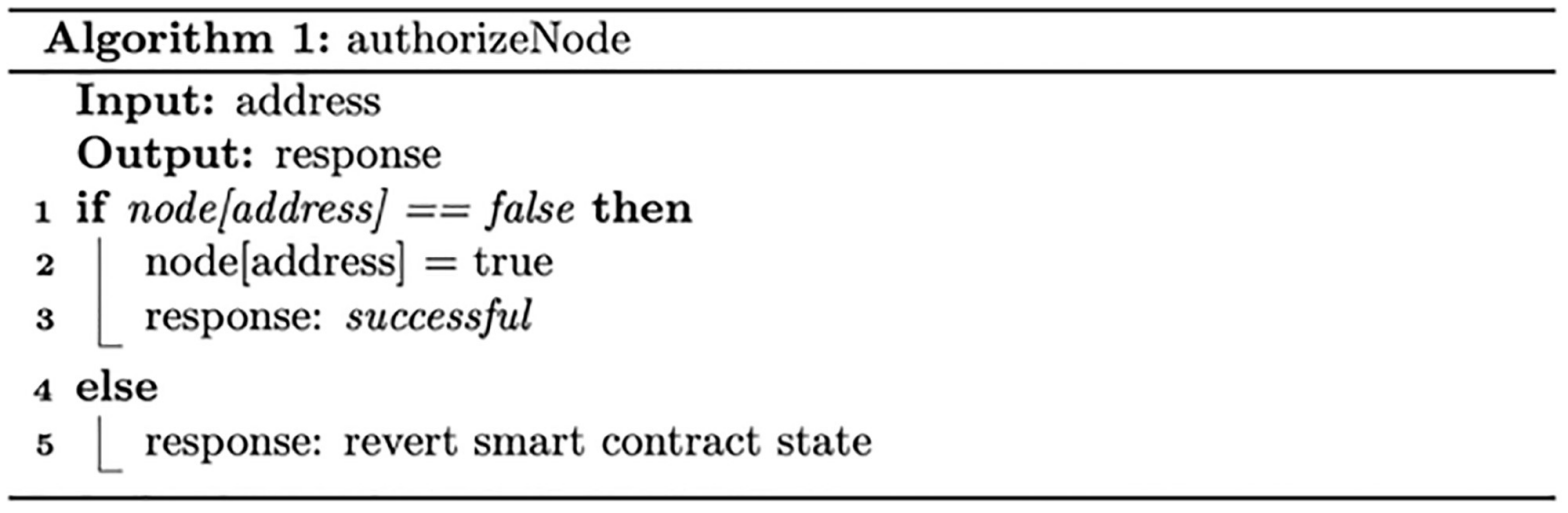
Node granting authorisation process.

**Fig 3:**
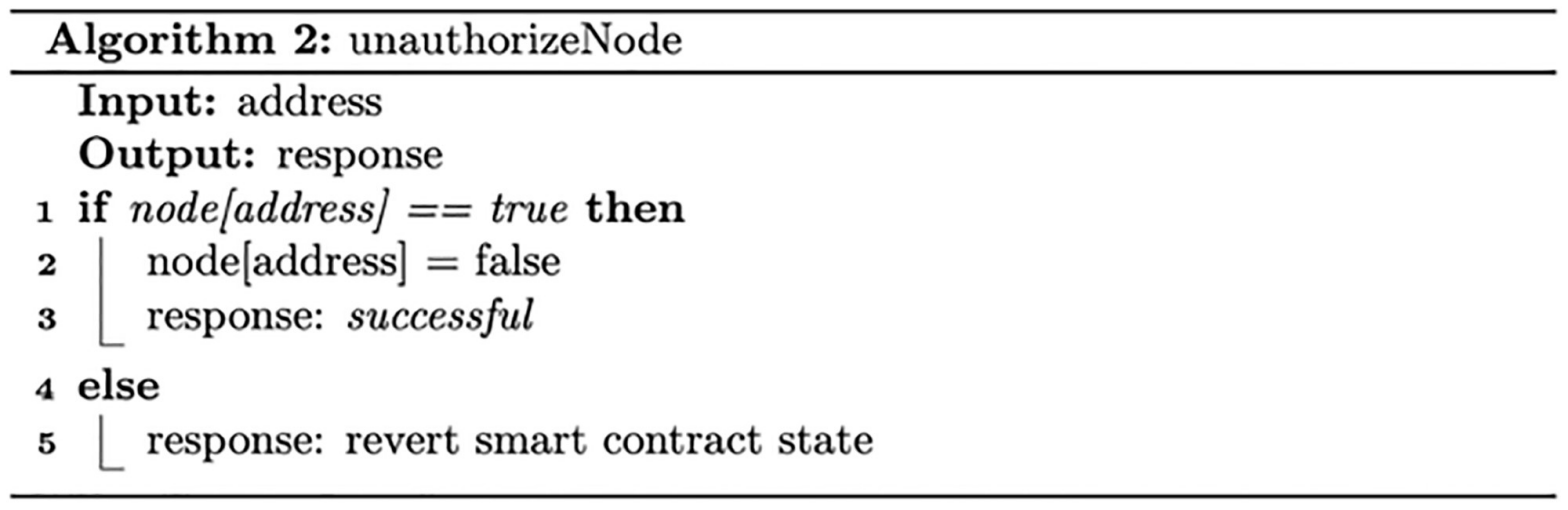
The revoking authorisation process for a given node.

Fig 2 describes the node granting authorisation process. Based on the node type, the node executes a specific smart contract function to request joining the system. The system admin, responsible for setting up the system and validating nodes’ registration requests, validates nodes’ identities via an off-chain process. Following successful validation, a specific smart contract function is executed to approve the granting authorisation request, which assigns a specific role to the node. Fig 3 describes the revoking authorisation for a given node. The system admin can authorise a given node by executing a specific function that changes the node status to unauthorised.

#### Trusted Behaviour Smart Contract and run-time behaviour smart contract

**Fig 4:**
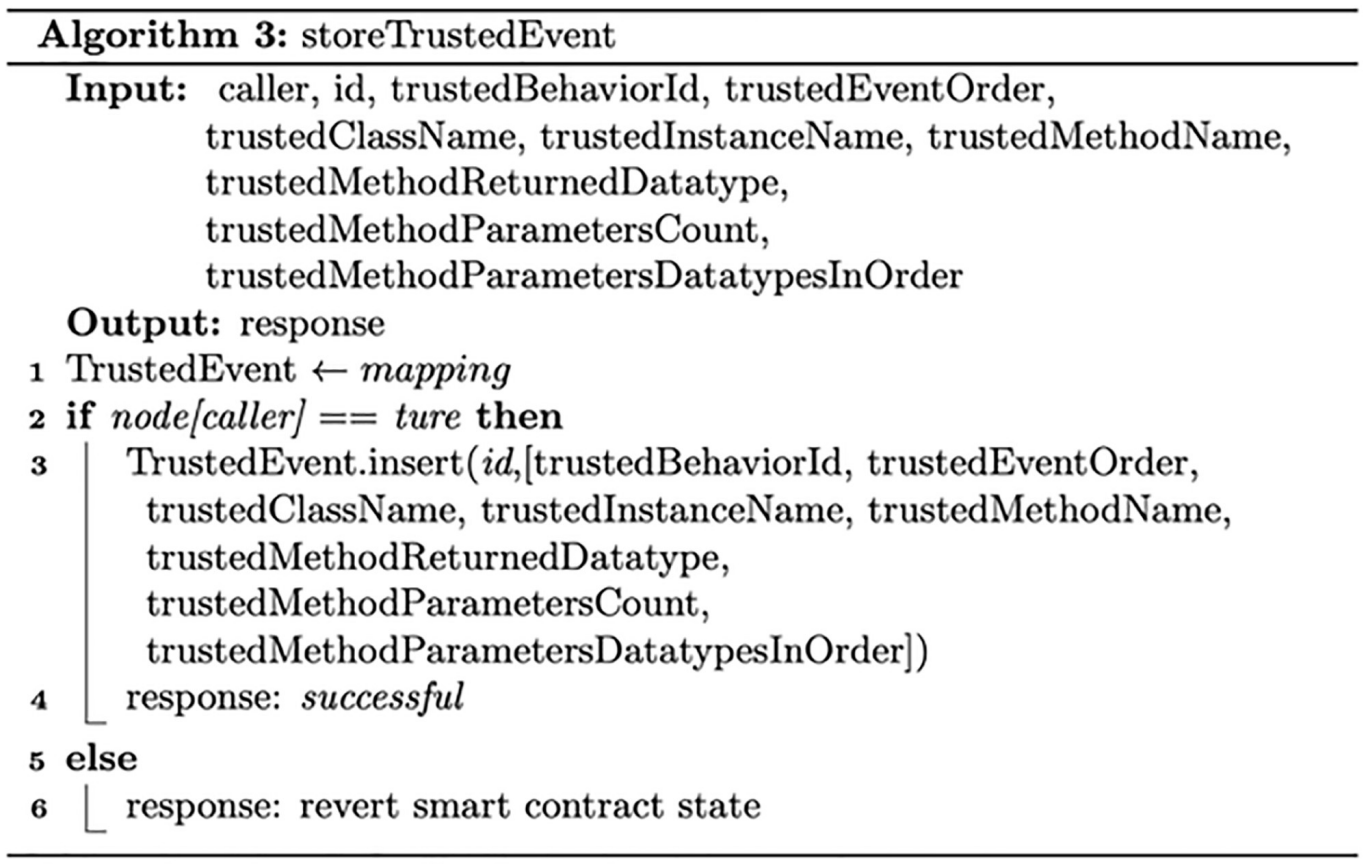
The process of storing and managing trusted software behaviour.

**Fig 5:**
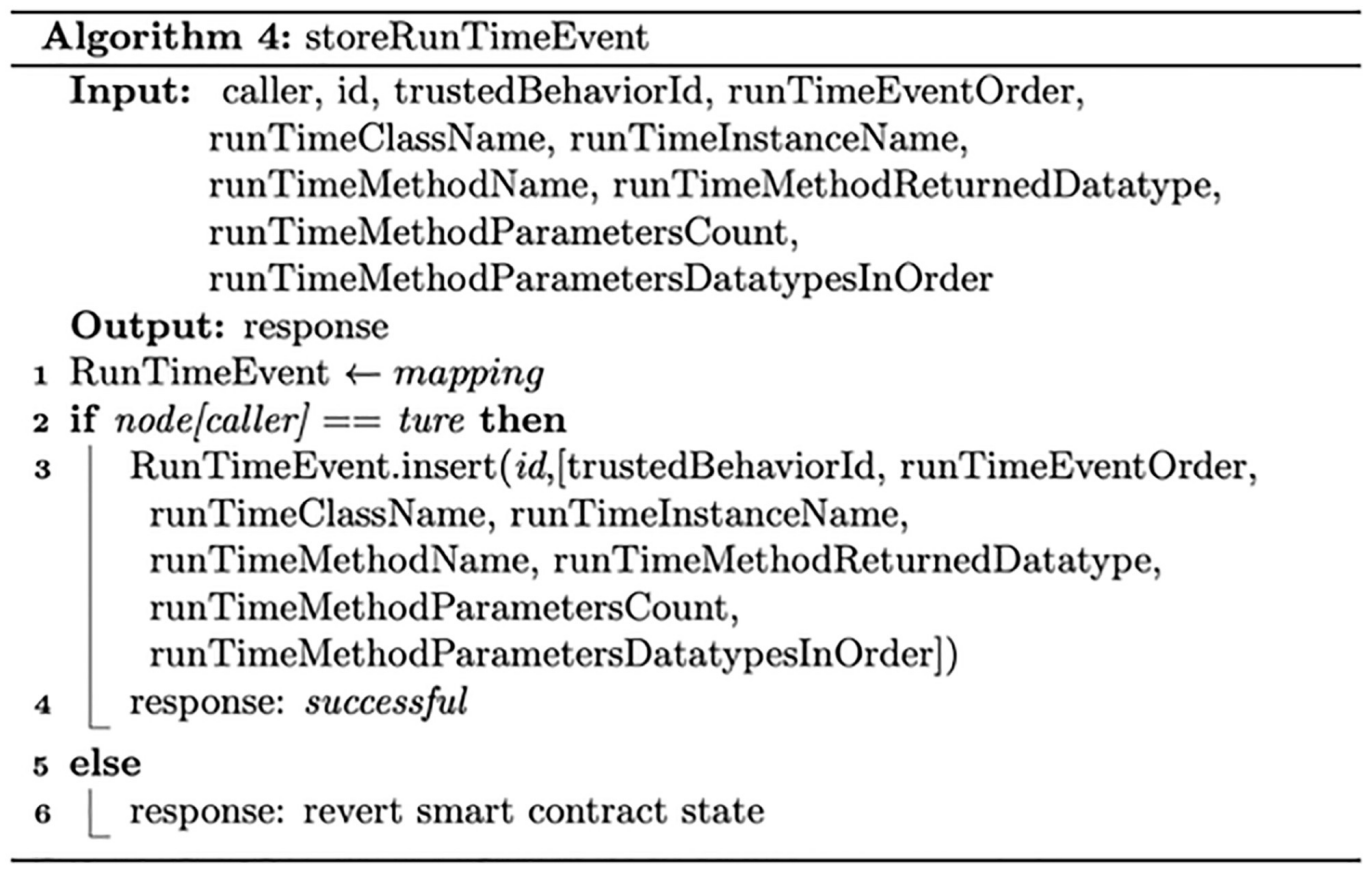
The process of storing and managing run-time software behaviour.

Figs 4 and 5 describe the process of storing and managing trusted software behaviour and run-time software behaviour, respectively. For efficient data retrieval and validation, trusted software behaviour and run-time software behaviour are each stored in a mapping data structure. A mapping is a hash table data structure that consists of key–value pairs. In the proposed system, the trusted software behaviour and run-time software behaviour each would be given an ID that would be stored as a key associated with a data structure that represents stored data.

## Results

### Proof of concept

It is vital to determine whether blockchain is the right solution for a given problem. According to the decision scheme of Wüst and Gervais [23] the use of blockchain in the proposed mechanism is justifiable. The software behaviour has to be stored as a state, there are multiple writers (software developer, software user and software tester), there is no Trusted Third Party (TTP), all writers are known, but some are not trusted, and finally, the software behaviour state should be publicly verifiable. Therefore, the proposed mechanism provides a clear technical rationale for using permissioned blockchains.

**Fig 6:**
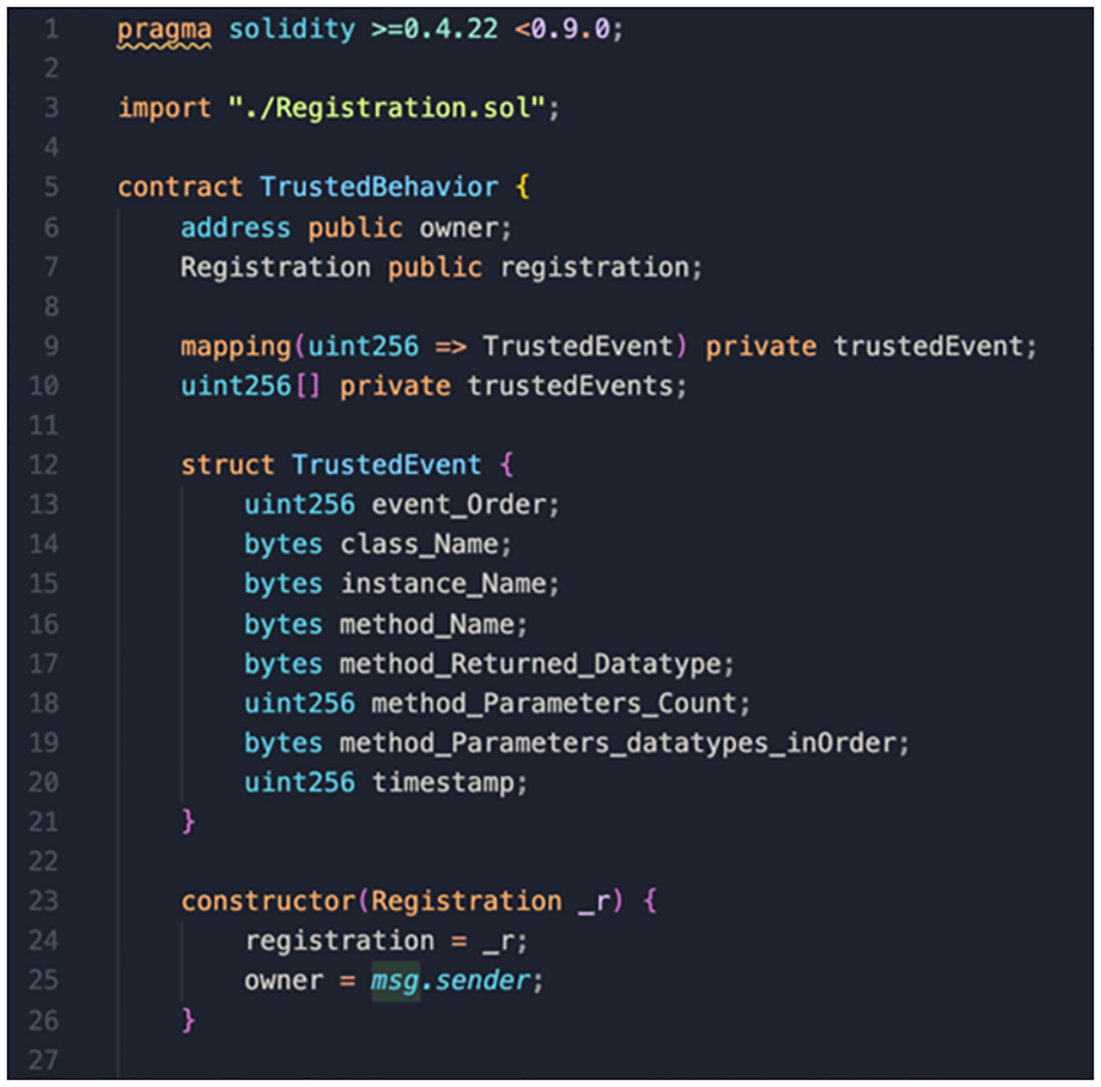
An example of trusted behaviour smart contract code.

**Fig 7:**
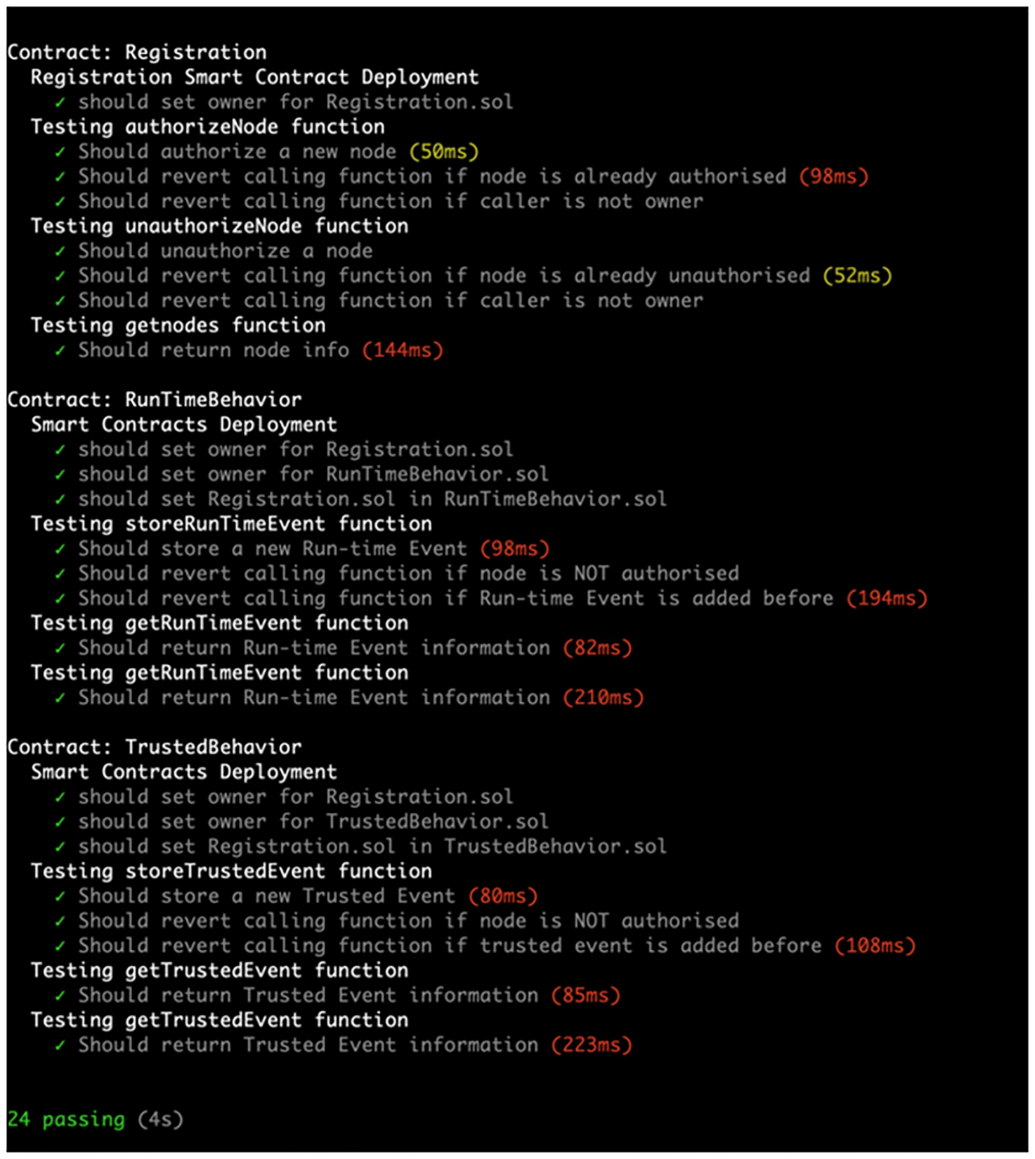
The result of smart contract testing units.

To demonstrate the feasibility of the proposed blockchain-based framework, we implemented a proof-of-concept on two permissioned blockchains. We used Hyperledger Besu to build level-1 and level-2 blockchains. The system smart contracts were written using the Solidity programming language, where the truffle framework, an Ethereum smart contract development tool, was used to test, compile and deploy system smart contracts. Fig 6 shows a portion of the *Trusted Behaviour Smart Contract* code, whereas Fig 7 shows the result of smart contract testing units. Lastly, we utilised Node.js to develop the off-chain resources, including *Sequence Diagram Generator*, *Behaviours Extractor*, *Design Recovery* and *Behaviours Comparator*.

**Fig 8:**
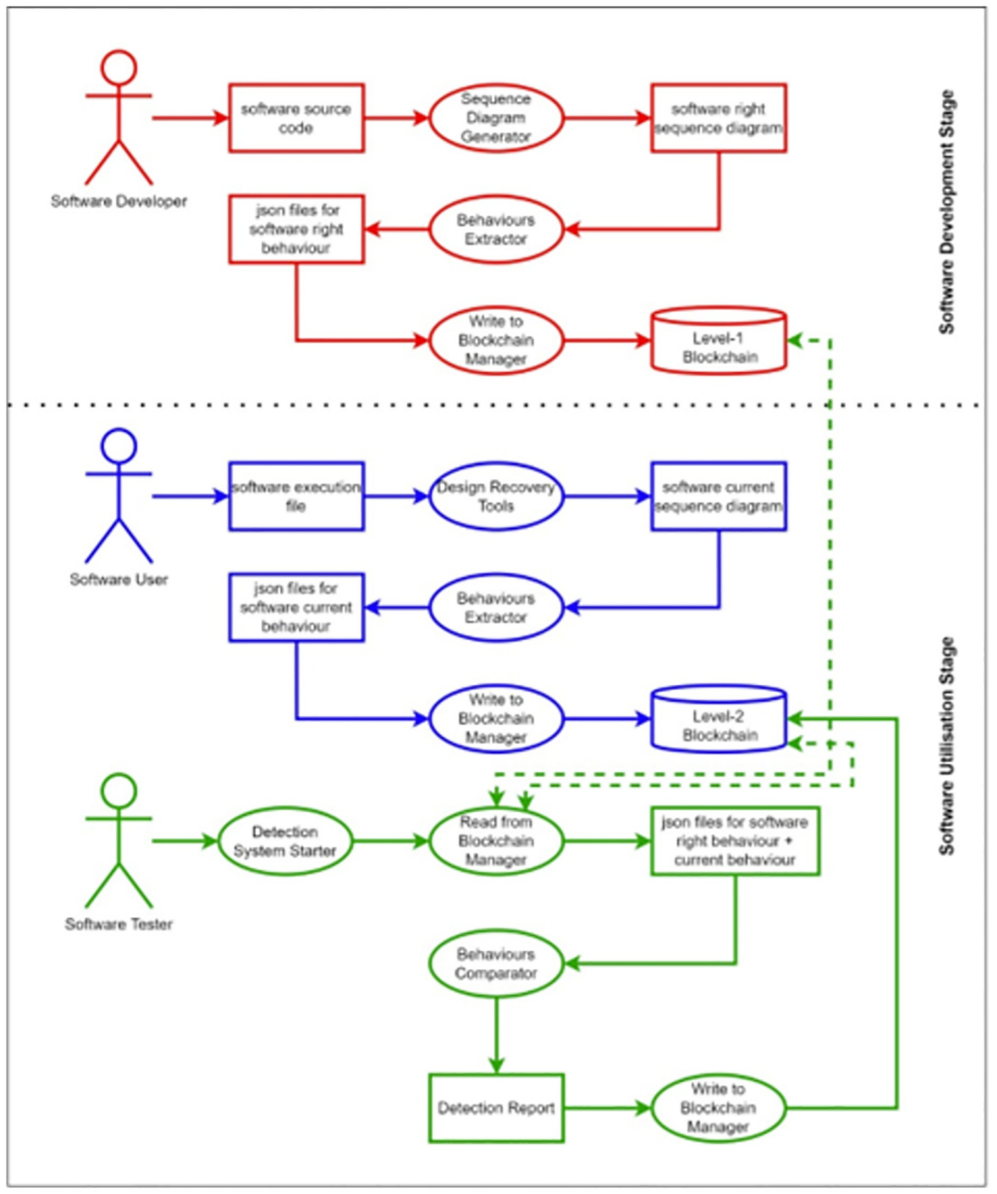
The high-level structure and workflow of the proof of concept.

The high-level structure and workflow of the proof of concept are shown in Fig 8. This figure shows the proposal design. It highlights that the proposal has to be performed through two stages: the software development stage and the software utilisation stage. In the first stage, the software developer or any other actor that owns a software source code converts the source code to a sequence diagram via any Sequence Diagram Generator tool. The obtained sequence diagram represents the right behaviour of the software as the sequence diagram is one of the UML behavioural models. However, the obtained sequence diagram needs to be manipulated to obtain the right behaviour of the software in an easy-to-use format. Hence, the Behaviours Extractor tool is proposed to simply convert the sequence diagram from the graphical and text-based representation to the JSON file representation, which is more readable and exchangeable. The obtained JSON file then needs to be stored on the blockchain, which should occur through a proposed Write to Blockchain Manager tool that is responsible for writing the right behaviour to the blockchain accurately and securely. The proposed blockchain for storing the right behaviour is notated as level-1 blockchain, which refers to the accessibility level for writing on this blockchain that is restricted to the software developers and the related actors to increase the trustiness in the stored behaviours. Reading from this blockchain, in contrast, has a less restrictive level due to the need to have more access to read from this blockchain, where the read will not affect the integrity of the data.

In the second stage, the software user or any actor that has the software execution file of the same software that has been dealt with in the first stage will have the ability to obtain the sequence diagram of the current software used by him/her. The obtained sequence diagram from the current software execution file is supposed to be identically the same as the sequence diagram obtained from the source code; otherwise, the current software execution file is considered to be affected by any potential attacks or damages. To get the sequence diagram from the current software, any Design Recovery tool can extract the sequence diagram during the execution time. Hence, the obtained sequence diagram can be converted to JSON files and then stored in blockchain–with less restriction to either write or read, notated as level-2 blockchain–in the same manner as occurred in the first stage.

The advantage of the proposal is most evident when the software tester–who can be the software user–wants to check the integrity of the software against any potential attacks or damages. The software tester via the proposed tool Read from Blockchain Manager reads both the right behaviour JSON files and current behaviour JSON files from their related blockchains. Then, via the proposed tool Behaviours Comparator, it checks that the current behaviour is consistent with the right behaviour in both the general behaviour layout and in the details of each event in the behaviour. A detection report is obtained from this tool that determines whether there is a potential attack on the current software or not, and this report is stored in the level-2 blockchain that is less restricted to read and write, which is compatible with the need for access to such as these types of reports.

### Performance evaluation

To evaluate the performance of the proposed framework, we utilised eight virtual machines running on Google Compute Engine (https://cloud.google.com) to provide the infrastructure for level-1 and level-2 blockchains. We used Hyperledger Besu (https://besu.hyperledger.org/), an open-source Ethereum client that provides permissioned blockchain networks. We also used an open-source benchmarking tool called Hyperledger Caliper (https://www.hyperledger.org/use/caliper) to evaluate the performance of the proposed framework. Table 2 shows the settings of the testing environment.

**Table 2:** Configurations of Hyperledger Besu and the testing environment.

Factor	Setting
**Nodes**	Eight virtual machines running on Google Cloud, where each virtual machine has a 2.7 GHz, 4-core Intel 7 CPU
**Peer-to-Peer Network**	Hyperledger Besu v22.4.4 3 validator nodes 5 peer nodes
**Consensus Protocol**	Clique
**Smart Contract Programming Language**	Solidity
**Benchmarking Tool**	Hyperledger Caliper v0.5.0

Two main types of blockchain operations, *Transaction* and *Query* operations, were tested using four performance metrics to evaluate the proposed framework: *Transaction throughput*, *Query throughput*, *Transaction latency* and *Query latency*. In this performance test, we focused on the main smart contract functions that represent the *Transaction* and *Query* operations of the proposed system (Table 3).

**Table 3:** Main smart contract functions of the system.

Main Function	Operation Type
authorizeNode	*Transaction*
unauthorizeNode	*Transaction*
getNode	*Query*
getNodes	*Query*
storeTrustedEvent	*Transaction*
getTrustedEvent	*Query*
getTrustedEvents	*Query*
storeRunTimeEvent	*Transaction*
getRunTimeEvent	*Query*
getRunTimeEvents	*Query*

We created a test module for each *Transaction* and *Query* operation. To reduce the likelihood of errors due to system overload and network congestion, tests were performed in multiple rounds with a fixed number of transactions and different send rates. We measured the *latency* and *throughput* for both operations *Transaction* and *Query* by changing the send rate from 100 to 1000 transactions per second (tps) within 10 testing rounds. Tables 4 and 5 summarise the experimental settings used for the *Transaction* and *Query* operations evaluation, respectively.

**Table 4:** Experimental settings for Transaction operations.

Test Number	1	2	3	4	5	6	7	8	9	10
**Functions Under Test**	Transaction operations									
**Worker Number**	1 worker									
**Transaction Number**	1000 transactions									
**Type of Control Rate**	Fixed rate									
**Send Rate (tps)**	100	200	300	400	500	600	700	800	900	1000

**Table 5:** Experimental settings for Query operations.

Test Number	1	2	3	4	5	6	7	8	9	10
**Functions Under Test**	Query operations									
**Worker Number**	1 worker									
**Transaction Number**	1000 transactions									
**Type of Control Rate**	Fixed rate									
**Send Rate (tps)**	100	200	300	400	500	600	700	800	900	1000

**Fig 9:**
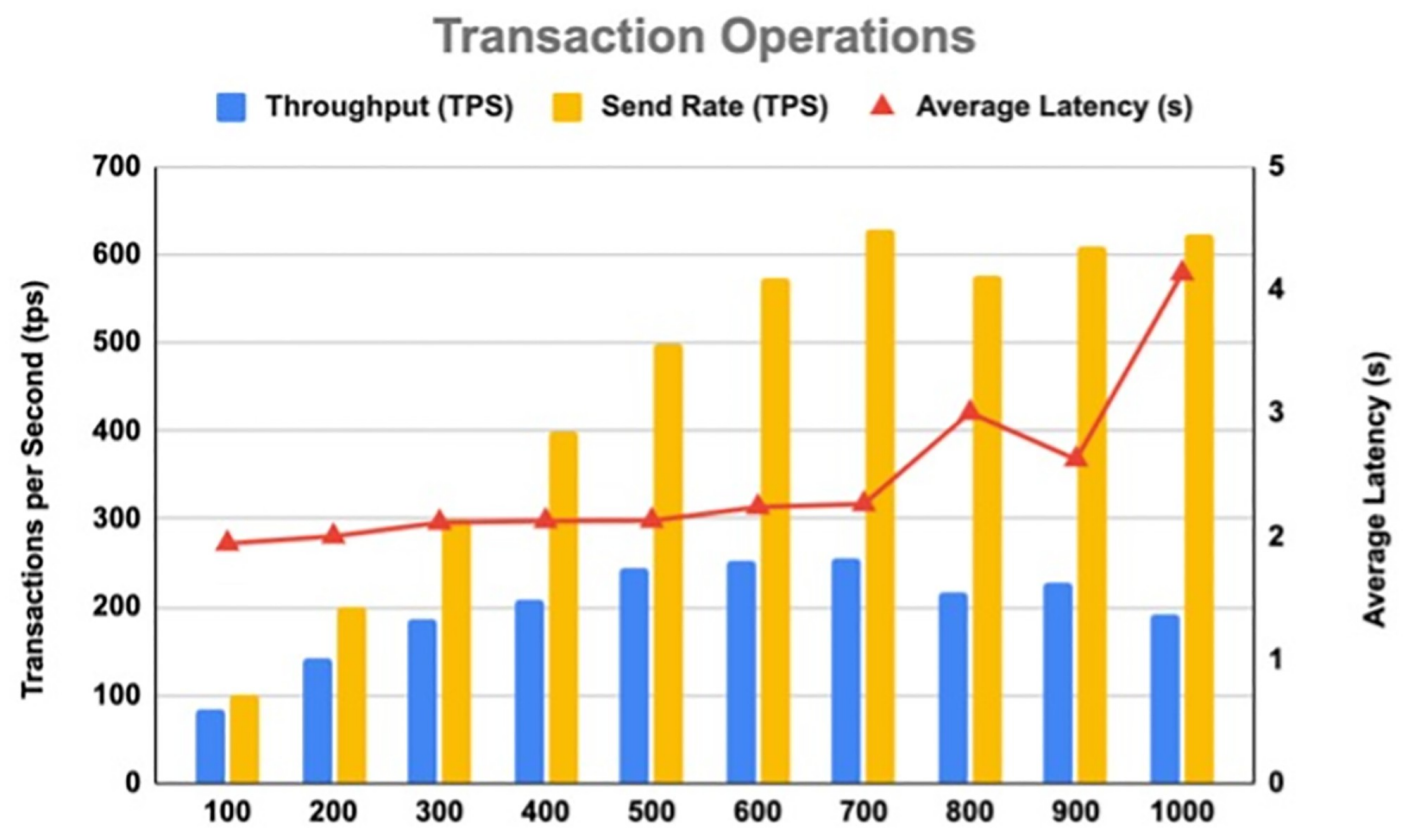
Throughput and latency for transaction operations.

**Fig 10:**
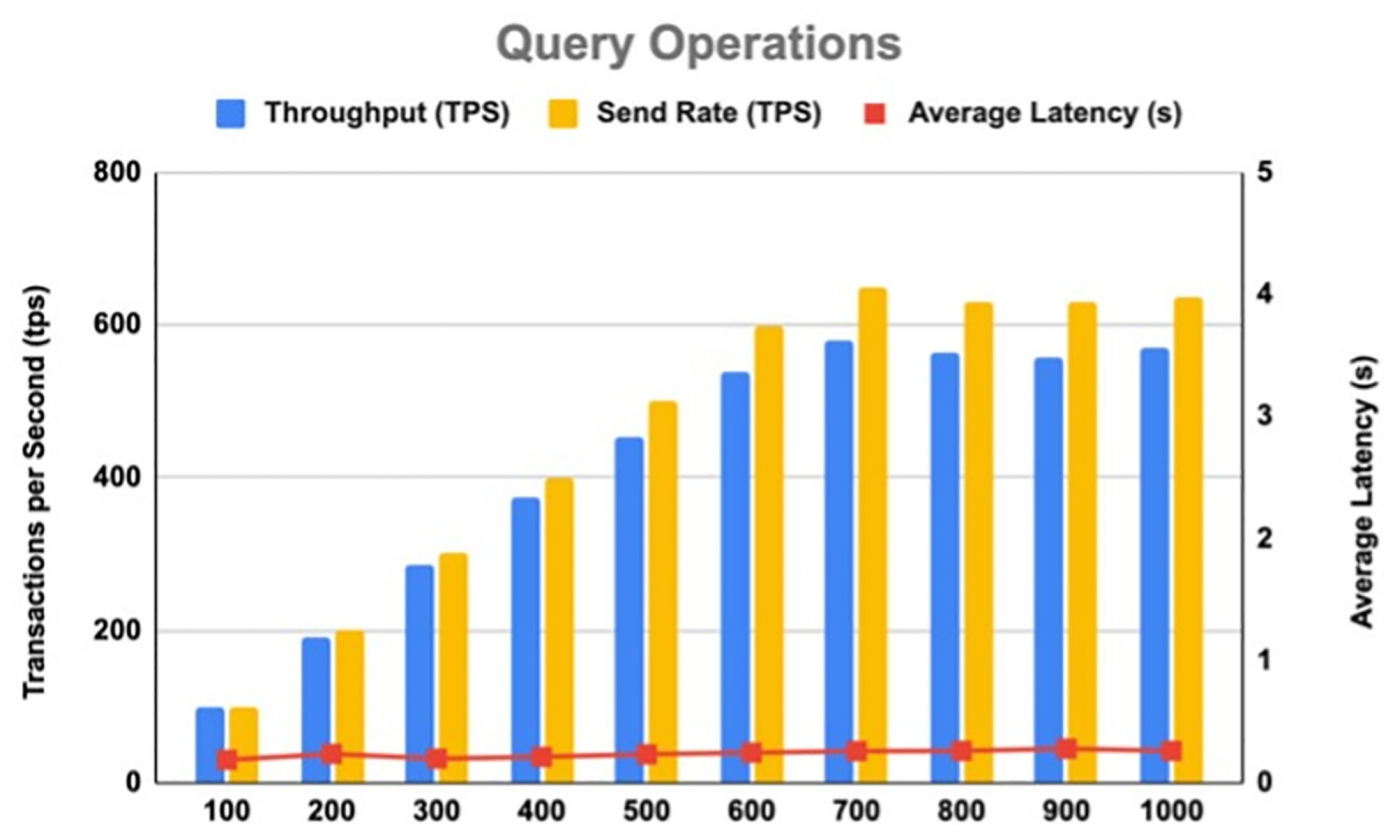
Throughput and latency for query operations.

Table 6 shows the results of the throughput and latency for Transaction operations, indicating an average throughput of 201.16 tps and an average latency of 2.46 s. Fig 9 illustrates the results of the throughput and latency for Transaction operations. On the other hand, Table 7 shows the results of the throughput and latency for Query operations, indicating an average throughput of 421.18 tps and an average latency of 0.18 s. Fig 10 illustrates the results of the throughput and latency for Query operations.

**Table 6:** Transaction operation results.

Test Round	Transaction Number	Transactions per Second (tps)	Send Rate (tps)	Max Latency (s)	Min Latency (s)	Average Latency (s)	Throughput (tps)
1	1000	100	100.13	2.59	1.40	1.95	83.10
2	1000	200	200.30	2.77	1.41	2.00	142.95
3	1000	300	300.43	2.78	1.45	2.12	186.78
4	1000	400	399.10	2.97	1.43	2.13	208.68
5	1000	500	498.55	2.89	1.48	2.13	244.15
6	1000	600	572.75	3.06	1.47	2.24	252.60
7	1000	700	628.25	3.06	1.46	2.27	256.98
8	1000	800	575.58	4.04	1.51	3.01	217.05
9	1000	900	609.70	3.70	1.59	2.63	226.80
10	1000	1000	624.50	5.69	2.01	4.14	192.55

**Table 7:** Query operation results.

Test Round	Transactions Number	Transactions per Second (tps)	Send Rate (tps)	Max Latency (s)	Min Latency (s)	Average Latency (s)	Throughput (tps)
1	1000	100	100.117	0.310	0.180	0.190	98.300
2	1000	200	200.233	0.453	0.180	0.238	189.933
3	1000	300	300.467	0.377	0.180	0.200	284.667
4	1000	400	400.700	0.405	0.180	0.213	373.067
5	1000	500	500.567	0.432	0.180	0.235	454.350
6	1000	600	599.667	0.453	0.180	0.248	539.900
7	1000	700	648.750	0.463	0.180	0.262	579.133
8	1000	800	629.883	0.472	0.180	0.263	564.133
9	1000	900	628.983	0.470	0.180	0.280	558.250
10	1000	1000	637.433	0.485	0.180	0.263	570.017

**Fig 11:**
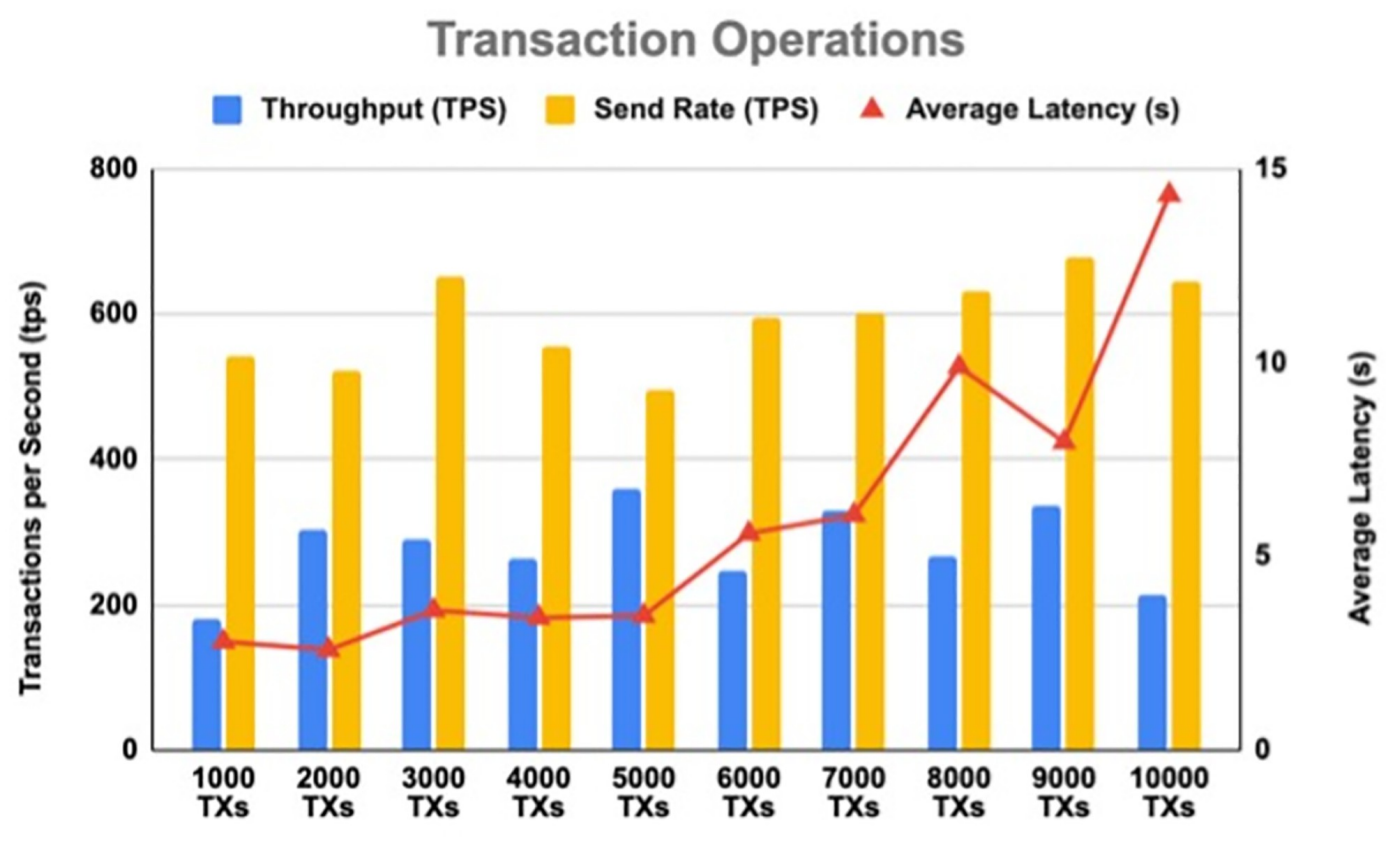
The impact of the increasing number of transactions on throughput and latency for Transaction operations.

**Fig 12:**
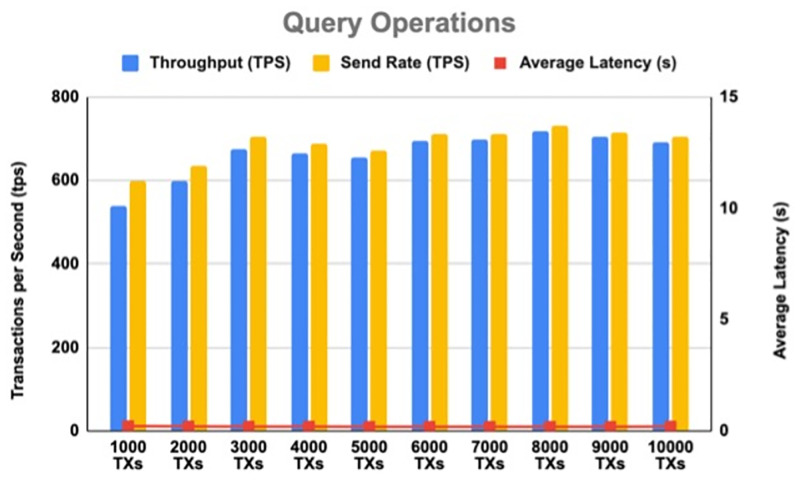
The impact of the increasing number of transactions on throughput and latency for Query operations.

To explore the system performance limits, the total number of transactions was selected as a parameter to measure the system to find the tipping point of the system. During the test rounds, the total number of transactions changed from 1000 to 10,000. Table 8 summarises the results of tests on system performance limits for *Transaction* and *Query* operations. Figs 11 and 12 illustrate the results of system performance limits for *Transaction* and *Query* operations, respectively.

**Table 8:** Results of the system performance limits.

Test Round	Transaction Number	Transaction Operations			Query Operations		
		Send Rate (tps)	Average Latency (s)	Throughput (tps)	Send Rate (tps)	Average Latency (s)	Throughput (tps)
1	1000	543.8	2.8	180.3	599.2	0.22	539.1
2	2000	520.7	2.59	305	634.7	0.21	599.2
3	3000	652.7	3.61	289.7	703.7	0.2	674.6
4	4000	555.4	3.42	265	687.6	0.2	666.4
5	5000	496.5	3.47	359	670.4	0.19	654.2
6	6000	595.8	5.59	247.2	710.6	0.19	695.3
7	7000	600.9	6.07	329.1	712.9	0.19	699.7
8	8000	632.2	9.89	267.1	729.9	0.19	717.7
9	9000	678.1	7.95	337.9	716.1	0.19	705.7
10	10000	645.4	14.33	213.2	704.8	0.2	690.9

As shown in Figs 11 and 12, the latency was flat for Query operations in all tests at about 0.19 s, while it was higher for Transaction operations between 2.8 and 14.33 s. When the number of transactions sent to the blockchain increases from 1000 to 5000 transactions, the Transaction operation latency increases slightly, but when it increases to more than 6000 transactions, the Transaction operation latency increases significantly. On the other hand, the Query average throughput is 690.9 tps, while the Transaction average throughput is 279.35 tps.

## Discussion

It is evident from the proposed framework and the proof of concept that this work applies a new technique. It is a security testing technique that extracts sequence diagrams from distributed software. These diagrams are used to determine whether attacks occurred. The proposed blockchain mechanism provides secure storage for the extracted sequence diagrams, compatible with their data structure. Sequence diagrams and blockchain blocks share a similar layout, ensuring compatibility. Hence, storage, consensus and retrieval mechanisms are expected to be straightforward and effective. Hence, we claim that the proposed framework provides a new and effective blockchain-based mechanism for detecting attacks.

## Conclusion

Using blockchain technology to test software security is a potent approach since software exhibits similar behaviour on different servers. Thus, code modification, deletion, or addition is complicated. Using the source code concerning the behaviour of an object-oriented software system to create a block is a novel approach that adds an additional layer of security. It is very hard to manipulate source code classes, methods, or variables because a matching instance exists in several servers.

## Supporting information

S1 File (ZIP)
